# Correlations between clinical features and death in patients with severe fever with thrombocytopenia syndrome

**DOI:** 10.1097/MD.0000000000010848

**Published:** 2018-06-01

**Authors:** Jianhua Hu, Siying Li, Xuan Zhang, Hong Zhao, Meifang Yang, Lichen Xu, Lanjuan Li

**Affiliations:** a State Key Laboratory for Diagnosis and Treatment of Infectious Diseases, Collaborative Innovation Center for Diagnosis and Treatment of Infectious Diseases, the First Affiliated Hospital, College of Medicine, Zhejiang University, Hangzhou; b Department of Infections, Hangzhou First People's Hospital, Nanjing Medical University, Hangzhou, Zhejiang Province, China.

**Keywords:** infection, risk factors, severe fever with thrombocytopenia syndrome

## Abstract

Severe fever with thrombocytopenia syndrome (SFTS) is an emerging high-fatality infectious disease caused by a novel phlebovirus belonging to the *Bunyaviridae* family. Thus, the independent predictors of death in this disease must be identified to improve the survival of affected patients.

A total of 25 hospitalized patients with SFTS virus infection were enrolled in our study, and their medical records and laboratory data were reviewed. The risk factors for death were examined by binary logistic regression.

The patient age was significantly higher in the deceased cases than in those who recovered (*P* = .020). Moreover, the occurrence of shock, respiratory failure, hemorrhagic manifestations, kidney dysfunction, and arrhythmia was significantly more common in the deceased cases than in the recovered cases (*P* = .016, *P* = .004, *P* = .005, *P* = .002, *P* = .038). Univariate binary logistic regression showed that shock, arrhythmia, and hemorrhage, as well as PCT, serum creatinine (Scr), and blood urea nitrogen (BUN) elevations, were the risk factors for death (odds ratio, OR 28.5, *P* = .015; OR 13.5, *P* = .027; OR 36, *P* = .008; OR 28.5, *P* = .015; OR 36, *P* = .008; and OR 76.0, *P* = .004). However, the BUN increase was the only independent risk factor for death indicated by multivariate logistic regression (OR 76.0, *P* = .004).

SFTS presents with a high fatality rate. When patients with SFTS manifest shock, arrhythmia, hemorrhage, PCT increase, and Scr and BUN elevations, especially BUN > 8.2 μmol/L, health care providers should be alerted and must administer early intervention to prevent the progress to death.

## 1 Introduction

Severe fever with thrombocytopenia syndrome (SFTS) is an emerging infectious disease caused by a novel phlebovirus called SFTS virus (SFTSV), which belongs to the *Bunyaviridae* family.^[1]^ The disease was first reported in 2010 in China, and then eventually identified in Korea,^[2]^ Japan,^[3]^ the United States,^[4]^ and India.^[5]^ SFTSV is mainly transmitted by tick bite, most frequently *Haemaphysalis longicornis*.^[1]^ However, person-to-person transmission through contact with blood or body fluid from infected patients also occurred in China.^[6–8]^

The clinical symptoms and laboratory abnormalities of SFTS are non-specific and includes fever, malaise, myalgia, nausea, vomiting, diarrhea, leukocytopenia, thrombocytopenia, and elevated serum hepatic enzymes.^[1,9–12]^ Some severe cases may develop hemorrhagic signs, neurologic symptoms, arrhythmias, pancreatitis, serious pneumonia, hypotension and shock, disseminated intravascular coagulation (DIC), multiple organ dysfunction (MOD), and even death.^[1,9–12]^

SFTS has an average case fatality rate of 12%, and even 30% in some areas.^[1,8,13]^ However, no licensed vaccine or pharmaceutical options are currently approved.^[11]^ Thus, determining the related risk factors for death and intervening early are important for reducing mortality in such patients. Previous research ^[9,10,14]^ reported that older age, neurologic symptoms, hemorrhagic manifestations, DIC, acute lung injury (ALI)/acute respiratory distress syndrome (ARDS) are the risk factors for fatality. However, some published studies assessed the risk factors for death by univariate analysis.

In the current study, we retrospectively analyzed the clinical data of patients with laboratory-confirmed SFTS in our hospital. We aimed to identify the independent predictors of death through univariate and multivariate analysis to improve the survival of patients with SFTS by effective intervention.

## 2 Methods

### 2.1 Patients

From January 2014 to April 2017, patients who presented with laboratory-confirmed SFTSV infection were enrolled in our hospital. The medical records were reviewed by a trained team of physicians and entered in duplicate into a computerized system. Analysis of the following information was included: demographics, clinical characteristics, laboratory results, treatment history, time between disease onset and arrival at our hospital, and hospitalization. All of the patients with laboratory-confirmed infection were divided into a recovered group and a deceased group depending on their clinical outcomes. The demographics, clinical characteristics, and experimental results were reviewed by a trained team of physicians and input in duplicates into a computerized system. Most of laboratory results were collected in our hospital medical records, a small part of them were collected from patients medical documents provided by other hospital. The experimental data from our hospital and other hospital were tested by the department of laboratory of our hospital and other hospital. (Table 1, Table 2, and Table 3).

**Table 1:**
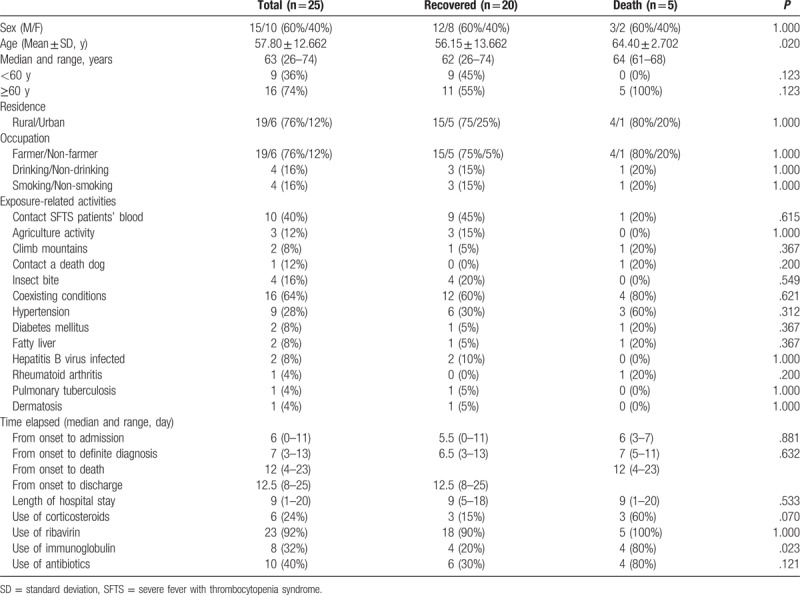
Characteristics of patients infected with severe fever with thrombocytopenia syndrome virus between recovered and death cases.

**Table 2:**
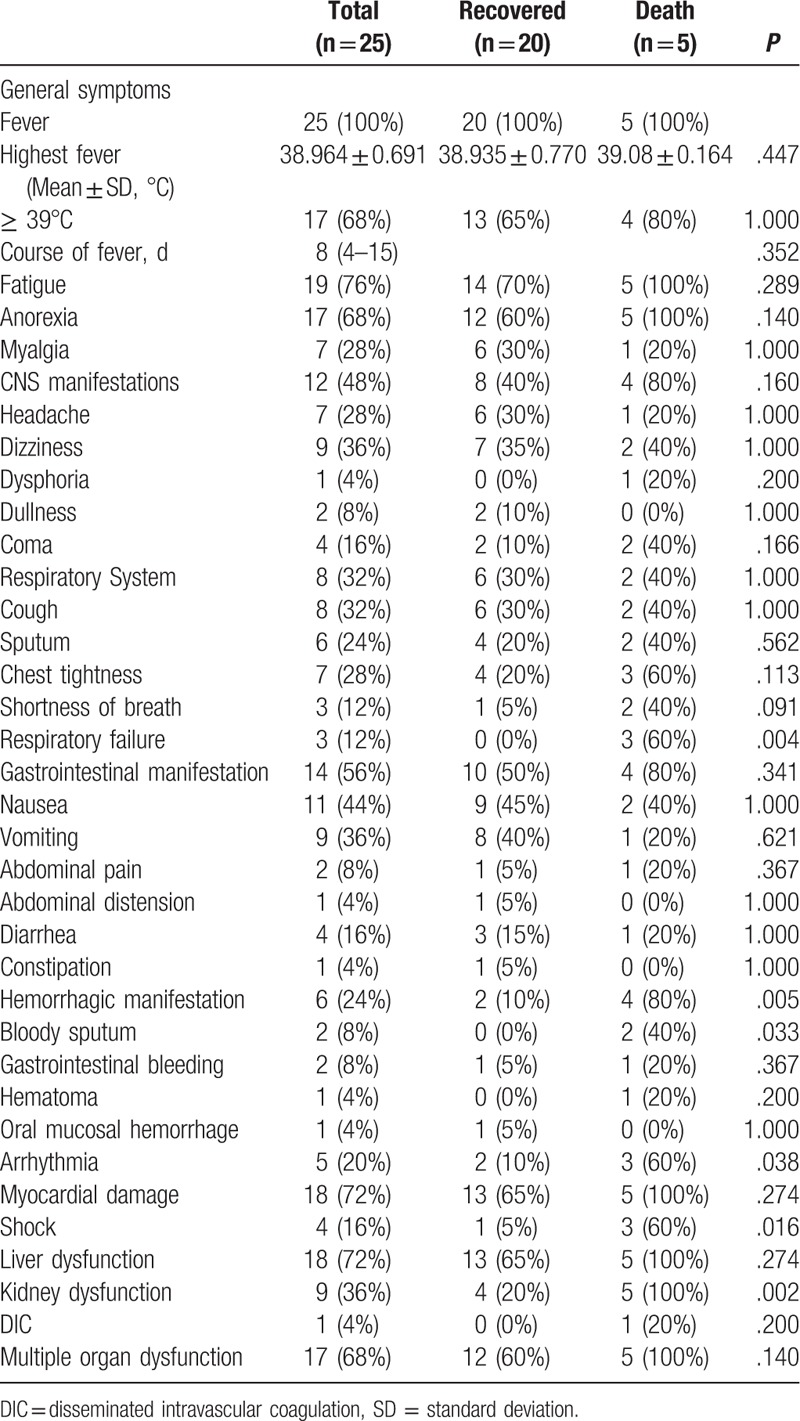
Clinical symptoms of patients infected with severe fever with thrombocytopenia syndrome virus between recovered and death cases.

**Table 3:**
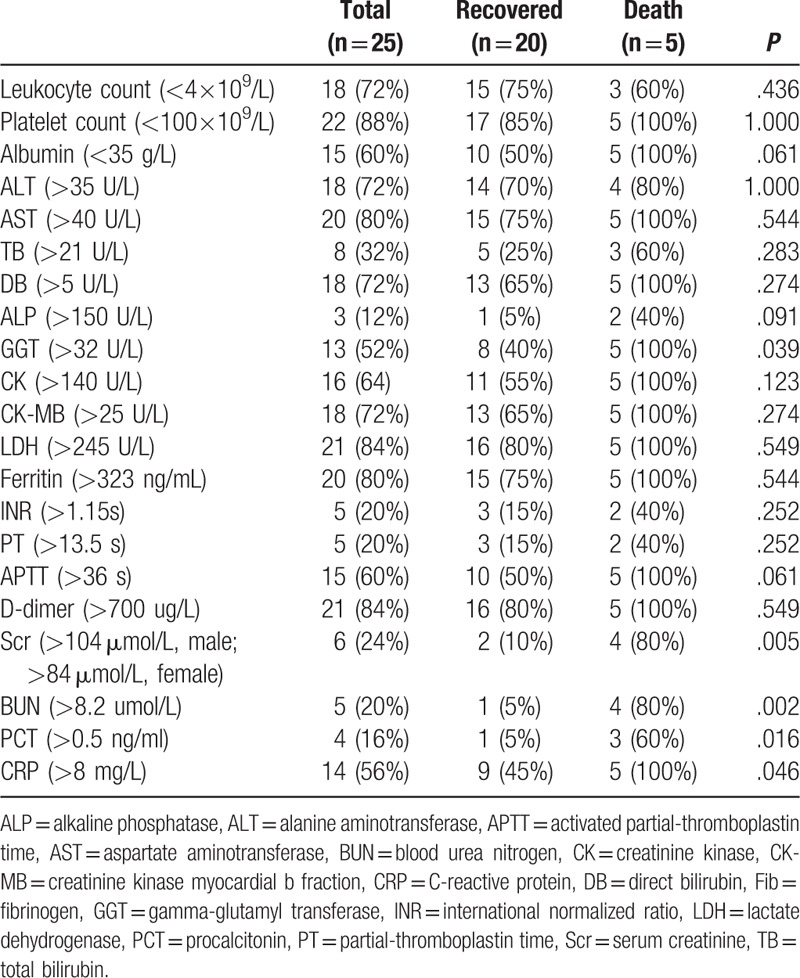
Laboratory features of patients infected with severe fever with thrombocytopenia syndrome virus during the course of illness.

### 2.2 Diagnostic criteria

According to the national guidelines,^[15]^ laboratory-confirmed SFTS should satisfy one or more of the following criteria: a positive SFTSV culture, a positive SFTSV RNA result by molecular detection, and seroconversion or fourfold increase in specific antibody to SFTSV between acute and convalescent serum samples.

Kidney dysfunction was defined as increased serum creatinine (SCr) and blood urea nitrogen (BUN) levels. Meanwhile, liver dysfunction was defined as elevated alanine aminotransferase (ALT) and aspartate aminotransferase (AST) levels and/or jaundice or even bleeding tendency. MOD was defined using criteria reported by Deitch.^[16]^

Shock was diagnosed on the basis of guidelines established in 2014.^[17]^ Respiratory failure was defined as arterial oxygen partial pressure (PaO_2_) below 8 kPa (60 mmHg) and/or carbon dioxide partial pressure (PaCO_2_) higher than 6.65 kPa (50 mmHg) at sea-level atmospheric pressure under resting conditions and breathing room air in the absence of a cardiac anatomic shunt and decreased primary cardiac output.

DIC was scored in accordance with the International Society on Thrombosis and Hemostasis scoring system.^[18]^ The scoring system included platelet count (>100 × 10^9^ cells/L, 0; < 100 × 10^9^ cells/L but >50 × 10^9^ cells/L, 1; and < 50×10^9^ cells/L, 2); elevated fibrin-related marker (D-dimer was used) (< 5000 ug/L, 0; < 9000 ug/L but ≥ 5000 ug/L, 2; ≥ 9000ug/L, 3); prolonged prothrombin time (< 3 s, 0; > 3 s but < 6 s, 1; and > 6 s, 2); and fibrinogen level (>1.0 g/L, 0; < 1.0 g/L, 1). A total score of ≥ 5 was considered compatible with overt DIC.

### 2.3 Statistical analysis

Statistical analyses were performed using the SPSS software (version 19.0, SSPS Inc., Chicago, IL). Results were expressed as the mean ± standard deviation (SD), median (range), and percentage. Means for continuous variables were compared using independent-group student *t* tests, for which the data were normally distributed; otherwise, the Mann-Whitney test was used. Categorical variables were analyzed by the chi-square test or the Fisher exact test. The risk factors for death in the patients were analyzed by binary logistic regression. All *P*-values were based on a 2-tailed test of significance (*P* < .05).

### 2.4 Ethics statement

Due to the retrospective nature of the study, informed consent was waived. However, the study was approved by the Ethics Committee of our hospital, the Medical ethics committee of the First Affiliated Hospital, College of Medicine, Zhejiang University, which conformed to the ethical guidelines of the Helsinki Declaration.

## 3 Results

### 3.1 Demographics

A total of 25 patients presented with laboratory-confirmed SFTS from January 2014 to April 2017. Fifteen patients were male, and 10 were female; 5 patients died, and the fatality rate was 20%. The mean age was 57.80 ± 12.66 years and the age range was 26 to 74 years. However, the age of the deceased cases was significantly higher than that of the recovered cases (*P* = .020).

Of the 25 patients, 16 (76.0%) were farmers living in rural areas. Meanwhile, 10 patients reported direct or indirect blood contact from a laboratory-confirmed SFTS before their disease onset. Moreover, 3 patients had been involved in agricultural activity, whereas 2 patients had climbed mountains, prior to disease onset. A patient came in contact with a deceased dog before disease onset. Among the patients, 4 had a confirmed history of tick bite of 1 day to 15 days prior to hospitalization, and included 1 patient bitten by tick during agricultural activity and another during mountain climbing. The transmission routes for the remaining 7 (28%) of 25 patients were unknown.

A total of 16 patients suffered comorbidities, namely, 7 with hypertension, 2 with concomitant hypertension and diabetes mellitus, 2 with fatty liver, 2 with hepatitis B virus infection, 1 with rheumatoid arthritis, 1 with pulmonary tuberculosis, and 1 with dermatosis (Table 1).

The median duration from symptom onset to hospital admission was 6 days (0–11 days), whereas that from symptom onset to diagnostic confirmation was 7 days (3–13 days). Moreover, the median duration from symptom onset until death was 12 days (4–23 days), whereas than until recovery (discharge) was 12.5 days (8–25 days). The median hospital length of stay was 9 days (1–20 days).

The differences in sex, residence, occupation, suspicious exposure history, coexisting conditions, time elapsed, smoking habit, and drinking habit between the recovered and deceased cases were not significant (Table 1).

Intravenous ribavirin was used in 23/25 (92%) of the patients. No significant difference was noted between the 2 patient groups. Of the patients, 6 (24%) used corticosteroids during their disease course, 8 (32%) used immunoglobulins, and 10 (40%) consumed antibiotics. However, most of the patients who used immunoglobulins were in the deceased group (*P* = .023) (Table 1).

### 3.2 Clinical manifestations

The clinical features are presented in Table 2. The most common clinical symptoms were fever (100%), fatigue (76%), and anorexia (68%), as well as gastrointestinal manifestations (56%), including nausea, vomiting, abdominal pain, abdominal distension, diarrhea, and constipation. All the patients suffered from fever during the disease course. Fever presented with a median duration of 8 days and ranged from 4 days to 15 days with a mean highest temperature of 38.964 ± 0.691°C. Of the patients that presented with fever, 68% became febrile beyond 39°C, and 48% manifested central nervous system symptoms, including headache, dizziness, dysphoria, dullness, and even coma.

Meanwhile, 32% suffered from respiratory symptoms, such as cough, sputum, chest tightness, shortness of breath, and even respiratory failure (16%). However, these patients were severe cases and finally ended in death.

Of the cases, 6 (24%) presented hemorrhagic manifestations, including bloody sputum (8%, 2/25), gastrointestinal hemorrhage (8%, 2/25), hematoma (4%, 1/25), and oral mucosal bleeding (4%, 1/25). Of these 6 patients, 4 progressed to death at a significant rate (*P* = .005), and 2 patients with bloody sputum also belonged to the deceased group. The recovered group did not include any patient with bloody sputum, the difference was significant (*P* = .033).

A total of 5 patients (3 from the deceased group and 2 from the recovered group) suffered from arrhythmia at a significant difference between the 2 groups (*P* = .038). Of the subjects, 72% presented with myocardial damage, 72% with liver dysfunction, 72% with kidney dysfunction, 68% with MOD, 16% with shock, and only 4% patients with DIC. Shock and kidney dysfunction occurred significantly more frequently in the deceased cases than in the recovered cases (*P* = .016, *P* = .002).

### 3.3 Laboratory features

The laboratory data for the recovered and deceased cases over the illness course are shown in Tables 3 and 4. Of the patients, 88% were noted with thrombocytopenia (platelets < 100 × 10^9^/L) even up to 9 × 10^9^/L and 78% with leukopenia (leukocyte count < 4 × 10^9^/L) reaching 0.5 × 10^9^/L. The deceased cases showed significantly lower platelet counts than those of the recovered group, and the median was 13 × 10^9^/L (46 × 10^9^/L for the recovered group) (*P* = .006). However, the difference in leukocyte count between the two groups was not significant (*P* = 1.000).

**Table 4:**
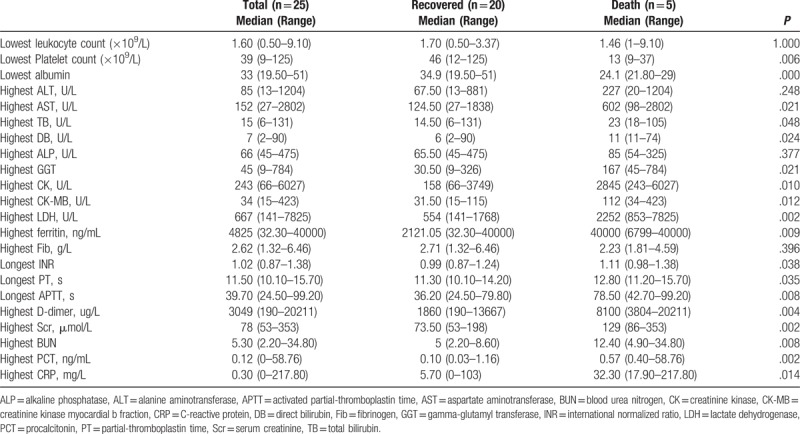
Differences in laboratory characteristics between recovered and death cases of severe fever with thrombocytopenia syndrome.

Most of the patients suffered from liver dysfunction and exhibited elevated ALT, AST, total bilirubin (TB), direct bilirubin (DB), alkaline phosphatase (ALP), and GGT levels, as well as decreased albumin levels. All of the deceased patients presented with increased GGT levels, whereas only 8 in the recovered group showed this abnormality; the difference between the two groups was significant (*P* = .039). For liver damage, the deceased cases presented with higher severity, as indicated by the higher AST (*P* = .021), TB (*P* = .048), DB (*P* = .024), and GGT (*P* = .021) levels than those of the recovered group.

Compared with the recovered cases, the deceased cases exhibited obvious kidney dysfunction; 80% of the decreased cases were recorded with Scr and BUN elevations (*P* = .005 and *P* = .002, respectively) and were higher in Scr and BUN levels (*P* = .002 and *P* = .008, respectively).

The deceased cases were also noted with elevated C-reactive protein (CRP) and procalcitonin (PCT) levels (*P* = .046 and *P* = .016, respectively) that were significantly higher than those of the recovered cases (*P* = .014 and *P* = .002, respectively).

Myocardial and muscle damage was more serious in the deceased cases than in the recovered cases with a median highest creatinine kinase (CK) level, highest creatinine kinase myocardial b fraction (CK-MB) level, and highest lactate dehydrogenase (LDH) activity of 2845 (243U/L-6027 U/L), 112 (34–423 U/L), and 2252 U/L (853–7825 U/L), respectively. The difference between the two groups was statistically significant (*P* = .010, *P* = .012, *P* = .002).

Furthermore, for blood coagulation, the deceased cases generally presented longer international normalized ratio (INR) (P = 0.038), prothrombin time (PT) (*P* = .035), and activated partial thromboplastin time (APTT) (*P* = .008), as well as higher D-dimer level (*P* = .004). We also found that the ferritin elevation in the deceased cases was greater than that in the recovered cases, with the highest ferritin level reaching 40000 ng/mL (*P* = .009).

### 3.4 Risk factors for death in patients

The risk factors for fatality in the patients, as determined binary logistic regression, are shown in Tables 5 and 6. Binary logistic regression of the univariate risk factors for death revealed that shock would increase death risk by 28.5 times (*P *= .015), arrhythmia by 13.5 times (*P *= .027), and hemorrhagic manifestations by 36 times (*P *= .008). PCT > 0.5 ng/mL enormously raised the death risk by 28.5 times (*P *= .015), Scr> 104 μmol/L for male sex and > 84 μmol/L for female by 36 times (*P *= .008), and especially BUN > 8.2 μmol/L by even 76 times (*P *= .004). However, binary logistic regression for multivariate risk factors for fatality showed that only high BUN (> 8.2 μmol/L) was the independent risk factor, thereby increasing the death risk by 76 times (*P *= .004).

**Table 5:**
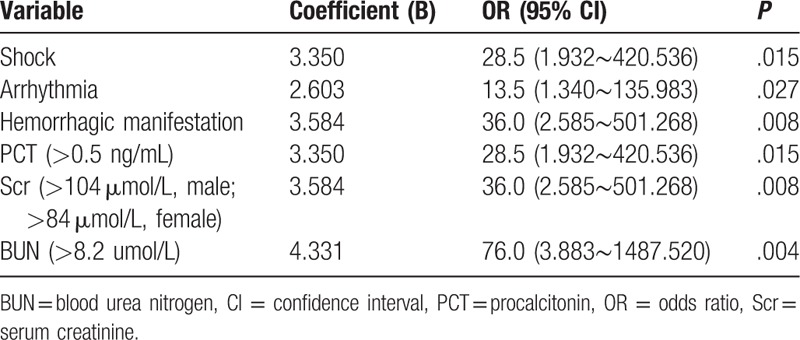
The univariate risk factors for the fatal patients infected with severe fever with thrombocytopenia syndrome virus infection.

**Table 6:**
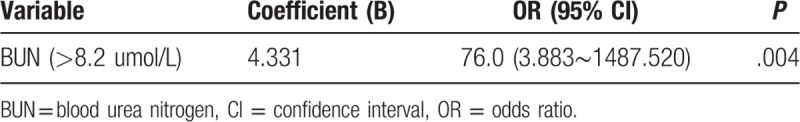
The multivariate risk factors for the fatal patients infected with severe fever with thrombocytopenia syndrome virus infection.

## 4 Discussion

We describe herein a cohort of 25 hospitalized patients with SFTSV infection and examined the risk factors for death by multivariate analysis. The fatality rate was 20%, similar to that in various studies.^[1,8,13]^ We found that fever, fatigue, anorexia, and gastrointestinal symptoms were the common clinical features of SFTS, as described previously.^[1,10,14]^ Deng BC et al^[14]^ reported that the major clinical syndromes in severe cases were disturbances of consciousness, arrhythmias, heart failure, ALI/ARDS, and DIC. They also reported that hemorrhagic manifestations, ALI/ARDS, and DIC were frequently observed in deceased cases.^[14]^ In the present study, besides hemorrhagic manifestation (including bloody sputum), we found that respiratory failure, arrhythmia, kidney dysfunction, and shock were more common in the deceased patients than in the recovered patients.

As previously verified, older age and prolonged delay from disease onset to hospitalization were found to be associated with fatal outcomes.^[10,19]^ Previous studies revealed that age is the critical risk factor or determinant of SFTS death.^[10,14,20,21]^ Similarly, the deceased patients in our study were more advanced in age than the recovered patients. The elderly individuals may have possessed low immunity to SFTSV and were hence more susceptible to SFTSV infection and likely easily progressed to severe disease and even death.

SFTSV infections could cause multiple system damage by direct or indirect mechanism, like most of other viruses. Clinical manifestations after its infection may include tissue injury, coagulation abnormalities, and acute-phase protein increases. Those who have severe organ dysfunctions had worse prognosis. In this study, we found that the deceased cases presented with lower platelet counts and albumin levels than those of the recovered cases. The deaths were also recorded with severe abnormalities in AST, TB, DB, GGT, CK, CK-MB, LDH, and ferritin levels than the recoveries. The deaths exhibited more prolonged INR, PT, and APTT; higher D-dimer, CRP, and PCT levels; and higher Scr and BUN levels. Serious systemic damage indicated poor prognosis. However, what cutoffs of laboratory data indicate poor prognosis must be determined. In the current study, we found that GGT > 32 U/L, Scr> 104 μmol/L (male) and Scr> 84 μmol/L (female), BUN > 8.2 μmol/L, PCT > 0.5 ng/mL, and CRP > 8 mg/L may predict poor prognosis.

To thoroughly understand the risk factors for death, we further analyzed the above suspicious factors by binary logistic regression (univariate and multivariate). Analysis revealed that shock, arrhythmia, hemorrhagic manifestation, PCT increase, and Scr and BUN elevations indicated poor prognosis. Unlike hemorrhagic manifestation, the other three risk factors were not reported until present. Studies ^[10,14]^ stated that melena was common among patients who eventually died, and no other hemorrhagic manifestation was substantively more common among those who died. Kidney dysfunction, expressed as Scr and BUN increases, was revealed as a risk factor for death by univariate analysis. However, unexpectedly, BUN increase was the only independent predictor of fatality determined by multivariate binary logistic regression. Possibly, when the SFTSV infects patients, protein catabolism increases, especially in patients with severe disease. However, further research is needed to validate this hypothesis.

As with all studies, our work has some limitations. First, there are only 25 cases in the study. Second, there will be some bias in retrospective study. Third, it is a big flaw that viral load was not included. Unfortunately, our hospital only conducted qualitative tests on SFTSV, and did not carry out quantitative detection. It is a retrospective study, and it is unable to further detect the viral load. However, here we aim to examine the SFTSV infections and the independent clinical predictors of death, qualitative value can meet the requirements of our research. We will address these drawbacks in our future studies.

In summary, our study results revealed that SFTS has a fatality rate of 20%, and the patients that progressed to death were generally older in age and prone to suffer from shock, respiratory failure, hemorrhagic manifestations (including bloody sputum), kidney dysfunction, and arrhythmia. These patients also tended to present with severe multiple-system damage, as indicated by serious abnormalities in related laboratory data (ie, platelet count; albumin, AST, TB, DB, GGT levels; CK, CK-MB, and LDH levels; ferritin levels; INR, PT, and APTT, D-dimer levels; CRP, and PCT levels; and Scr and BUN levels). However, shock, arrhythmia, hemorrhagic manifestation, PCT increase, and Scr and BUN elevations indicated poor prognosis for SFTS infection. Notably, BUN increase was the only independent risk factor for death revealed by multivariate analysis. Thus, when patients present with severe multiple-system damage, especially BUN > 8.2 μmol/L, health care providers should be alerted and must administer early intervention (such as immunoglobulin using and the corticosteroid impulse therapy) to prevent death.

## Acknowledgments

The authors would like to thank all of the participants recruited in this study.

## Author contributions

**Data curation:** Xuan Zhang.

**Formal analysis:** Hong Zhao.

**Funding acquisition:** Meifang Yang.

**Investigation:** Siying Li, Xuan Zhang.

**Methodology:** Siying Li, Lichen Xu.

**Project administration:** Lanjuan Li.

**Resources:** Hong Zhao.

**Writing – original draft:** Jianhua Hu, Lanjuan Li.
